# Synthesis and Characterization of Maguey (*Agave cantala*) Nano-Modified Bioplastic

**DOI:** 10.3390/polym18030325

**Published:** 2026-01-26

**Authors:** Kendra Felizimarie P. Magsico, Lorenz Inri C. Banabatac, Claudine A. Limos, Nolan C. Tolosa, Noel Peter B. Tan

**Affiliations:** 1 Center for Advanced New Materials, Engineering and Emerging Technologies (CANMEET), University of San Agustin, Iloilo City 5000, Philippines; kfmagsico@usa.edu.ph (K.F.P.M.); libanabatac@usa.edu.ph (L.I.C.B.); climos@usa.edu.ph (C.A.L.); ntolosa@usa.edu.ph (N.C.T.); 2 Department of Chemical Engineering, College of Technology, University of San Agustin, Iloilo City 5000, Philippines

**Keywords:** *Agave cantala*, acetylation, bioplastic, cellulose, cellulose acetate, maguey, regeneration

## Abstract

The environmental threat posed by small, single-use sachets sourced from 48% annual waste from excessive packaging has been assessed by investigating the development of nano-incorporated bioplastic films from the high-yield plant, maguey (*Agave cantala*). Maguey cellulose was acetylated (using 10 and 15 mL of acetic anhydride for 16, 24, and 32 h), successfully yielding a high of 81.34% maguey cellulose acetate (MCA). MCA was confirmed to contain acetate groups (C=O, C-H, C-O) via FT-IR and exhibited a hydrophobicity of a 121.897° contact angle. Bioplastic films were fabricated using MCA solution combined with 15% (*w*/*w*) commercial cellulose acetate (CCA)/MCA and reinforced with nanoclay (NC) at 0.5%, 1%, and 3% (*w*/*w*) concentrations. Nanomaterial incorporation generally improved properties; however, mechanical strength declined with increasing NC concentration, recording tensile strengths of 2.01 MPa, 0.89 MPa, and 0.78 MPa for the 0.5%, 1%, and 3% NC films, respectively. Conversely, the 3% NC film showed the best barrier property, with a water vapor transmission rate (WVTR) of 31.14 g/m^2^ h. Surface morphology confirmed NC integration (nanomaterial sizes 29.74 nm to 107.3 nm), and the 0.5% NC film displayed the smooth structure ideal for sustainable packaging. The slight increase in contact angle observed between the 0% NC (60.768°) and 0.5 NC (62.904°) films suggested limitations in NC dispersion. Overall, the findings demonstrate the potential of using regenerated maguey cellulose acetate to create nano-bioplastic films with tailored mechanical and barrier properties for sustainable packaging, though optimization of NC loading and dispersion is necessary to maximize strength.

## 1. Introduction

The Philippines is highlighted by the World Wide Fund for Nature (WWF) as a major contributor to marine plastic pollution because a large portion of the plastics is mismanaged. Only 9% of plastic waste is recycled, while 35% subsequently leaks into open environments, like waterways and the ocean, making the Philippines one of the world’s worst plastic offenders. A significant driver of this is packaging, which accounts for 48% of plastic production [1] and is the main source of marine litter. This problem is exacerbated by the country’s status as a “sachet economy”, where products are predominantly sold in single, one-portion packages, a common practice in developing nations. These small, non-recyclable sachets [2] are particularly alarming, comprising 52% of the residual plastic waste stream. They accumulate in the environment, polluting landscapes, choking waterways, and threatening wildlife, marine life, and livelihoods. These single-use sachets, found everywhere from small *sari-sari* stalls to large shopping centers, often contain surfactant-based products (e.g., soaps, detergents, and condiments). Filipinos frequently purchase them for their convenience, practicality, affordability, and availability [2]. The surfactant-based industry alone in the Philippines consumes an extraordinary amount, using no less than 1,560,000 tons of plastic materials for sachet packaging.

Commercial plastic packaging, made from non-renewable, non-biodegradable synthetic petrochemical polymers, poses significant environmental threats [3,4,5]. Addressing this issue requires adopting a circular economy model by redesigning packaging to minimize waste and promote resource regeneration. One solution involves developing alternative packaging material from natural polymers or bio-based thermoplastics, such as starch, cellulose, chitosan, pectin, polylactic acid (PLA), and polyhydroxyalkanoates (PHAs). However, many bio-based materials face challenges; for example, starch may compete with food demands, and biopolymers, like chitosan and pectin, are relatively expensive. Moreover, these natural bioplastic films typically lack the durability of conventional plastics, necessitating ongoing studies into biopolymer modification and enhancement [6]. Among natural polymers, cellulose is highly desirable. As the largest organic carbon reservoir on Earth, it is an abundant, renewable, and sustainable resource derived from agricultural waste [7,8,9,10]. Utilizing renewable sources like biomass and bio-waste creates value and drives the proactive use of locally available resources. Despite cellulose’s abundance, the Philippines currently lacks a notable packaging industry that utilizes bioplastic packaging.

Maguey (*Agave cantala*) is a fiber-abundant plant from the *Agavaceae* family, originating in the Yucatan Peninsula, Mexico. In the Philippines, it is cultivated in the north and central parts of the country. In Cebu alone, it is found in Aloguinsan, Cebu. Maguey fibers have outstanding properties such as quality tensile strength and modulus, high durability, low bulk density, good moldability, and recyclability. Other characteristics of maguey fiber include high tear resistance, high porosity, and high folding endurance. The leaves are long and drooping rosettes as they mature, containing highly valuable cellulose content of approximately 53.56% [7]. A preliminary three-stage footprint analysis considers the following: (1) maguey cultivation, emphasizing its inherent benefits, such as lower water consumption and arid land suitability compared to high-irrigation feedstocks [11,12]; (2) the chemical processing stage, utilizing data from analogous lignocellulosic bioplastic syntheses to identify key energy and material hotspots that will be the focus of future life cycle assessments (LCAs) and process optimization [13]; and (3) the disposal (end-of-life) stage, comparing the expected Global Warming Potential (GWP) of our novel material under ideal composting conditions versus conventional landfilling [14]. This step provides the essential environmental context needed now while committing to a full, dedicated LCA once the process is scaled to a pilot level.

The first publication to study maguey (*Agave cantala*) fiber for cellulose extraction and later for nanocellulose was carried out by Sumarago et al. in 2024 [8]. Cellulose yield of 53.56% was reported after alkali treatment and bleaching processes. Such cellulose yield of maguey is competitive with other *Agave* plant species that underwent similar processes. However, cellulose-based bioplastic, by itself, has poor mechanical properties, limiting its potential for bioplastic films. Many available fibrous alternatives are overlooked due to their excessive cellulose content that decreases mechanical strength [15,16]. The reinforcement of nanomaterials into bioplastic films aids in the improvement of properties, such as biocompatibility, mechanical strength, and thermal stability, and reduces water vapor permeability [17]. Most biodegradable polymers have “performance and processing” issues, and nanomaterials are used to improve the biomaterial’s performance. These bio-nanomaterials are nano-sized, less than hundreds of nanometers, and are used for reinforcement and functional enhancements [18,19,20]. Nanofillers are incorporated into the polymer matrix to improve or adjust material properties such as mechanical, thermal, and electrical–optical properties. Examples of these nanomaterials used for nanocomposites are nanoclay, carbon nanotubes, organic nanofillers, etc. [21,22]. Nanoclays are layered nanosilicates that have been developed as reinforcement fillers because of their novel properties for new packaging materials, such as high tensile modulus, strength, surface area, and high aspect ratio. Another aspect of interest in using nanoclay, other than its exceptional mechanical properties, is the incorporation of active ingredients that extend food shelf life and quality [23]. A study by Li et al. in 2018 reported an increase in bioplastic tensile strength from 48.96 MPa to 64.75 MPa with 5 wt.% of nanoclay montmorillonite. However, it was notably susceptible to unavoidable agglomeration [24]. Carbon nanotubes are super-strong and super-stiff polymer composite nanomaterials ideal for high-strength polymer composite applications, and they have been reported to increase Young’s modulus, thermal, electrical, and barrier properties of a PLA bioplastic material [25]. Cellulose nanocrystals are an organic nanofiller that increases bioplastic stiffness because of their nanoscale dimension, low density, high surface area of ≥100 $m^{2}/g$, >100 high aspect ratio, high crystallinity, and inherent rigidity [26,27]. A study by Xu et al. reported a higher tensile strength from a 10 vol.% reinforced cellulose nanocrystal-loaded starch film. The tensile strength successfully improved by 129% compared to that without nano-reinforcement; additionally, water vapor permeation was reduced by 17% [28,29]. Chitosan is also an organic nanofiller derived from the deacetylation of chitin from shell waste and skeletal material of insects and crustaceans. A study by Lopez, O. et al. in 2014 on thermoplastic corn starch (TPS) films showed that 10% (*w*/*w*) chitosan–starch reduced water vapor permeability by 35%, improved tensile strength by 17%, and improved elastic modulus by 13% [29,30].

The production cost of bioplastics is generally higher than that of conventional, fossil fuel-derived plastics, posing a primary barrier to mass market adoption. This disparity is rooted in the imbalance of economies of scale; conventional plastics benefit from decades of technological optimization and massive, globally established production infrastructure, which drives the per-unit cost remarkably low [31]. In contrast, bioplastics, such as polylactic acid (PLA), often rely on more expensive feedstocks (e.g., agricultural products, like corn or sugarcane), whose prices are tied to volatile commodity markets, unlike the deeply entrenched, large-scale petrochemical supply chains [32]. Production processes for bioplastics, which often involve complex fermentation and polymerization, are also generally less energy- and cost-optimized than the mature refining and polymerization processes for fossil fuel plastics [33]. Preliminary cost–benefit analyses indicate that the manufacturing price of bio-based PET can be in the range of USD 2.00–$3.00 per kg, while petrochemical PET remains lower, approximately USD 1.00 to USD 1.30 per kg, highlighting a significant economic disadvantage [34].

Scalability challenges for bioplastics span technical, logistical, and systemic hurdles. The reliance on bio-based feedstocks introduces issues of food vs. fuel/material competition and requires careful management of land and water use, which can result in trade-offs, like increased eutrophication or land use impacts, when production is scaled up [33,34]. Logistically, the bioplastic supply chain is less developed and more fragmented than the conventional plastic supply network, hindering consistent supply and quality at high volume [31]. Furthermore, the lack of sufficient industrial composting infrastructure and the risk of contamination when bioplastics are mixed into conventional recycling streams present significant systemic challenges. These issues prevent bioplastics from realizing their intended end-of-life benefits at scale and necessitate considerable upfront investment in specialized waste management and processing capacity to support market growth [31,34]. Ultimately, scaling requires comprehensive policy changes and financial incentives to close the cost gap and build the necessary infrastructure [11].

Developing locally sourced nano-modified bioplastic materials from the Philippines and investigating the critical fabrication parameters are essential at this stage. The research specifically sought to determine bioplastic fabrication ratios by testing maguey (*Agave cantala*) cellulose acetate against commercial cellulose acetate at concentrations of 15%, 25%, 35%, and 50% (*w*/*w*) and the effect of nanoclay incorporation at concentrations of 0.5%, 1%, and 3% (*w*/*w*). The final objective was to evaluate the mechanical properties of the resulting nano-incorporated regenerated cellulose bioplastics. The study was carried out to establish a clear proof of concept for successfully integrating two novel bio-additives: (1) maguey fiber for its inherent potential for biodegradability and its bio-derived composition and (2) nanoclay (NC) within the CCA-MCA bioplastic matrix. Given that this is the first report on this specific material combination, the scope was deliberately limited to establishing fundamental feasibility and identifying the optimal NC loading range.

## 2. Materials and Methods

Naturally retted maguey fibers were retrieved from the *Agave cantala* plant species, also known as “maguey” and locally found at Aloguinsan, Cebu. Prior to acetylation, maguey cellulose was isolated from maguey fibers. The protocol adopted for the maguey cellulose extraction was taken from Sumagaro et al.’s group [8]. The chemical reagents used in the experiment included acetic anhydride (≥99% (CH_3_CO)_2_O, Sigma-Aldrich, Merck Inc., Taguig City, Philippines), glacial acetic acid (100% CH_3_CO_2_H, Sigma-Aldrich, Merck Inc., Philippines), sulfuric acid (95–97% H_2_SO_4,_ Sigma-Aldrich, Merck Inc., Philippines), cellulose acetate (Average M_n_ ~30,000 by GPC%, Sigma-Aldrich, Merck Inc., Philippines), acetone (ACS Reagent CH_3_COCH_3_, Sigma-Aldrich, Merck Inc., Philippines), ethanol (ACS, ISO, Reagent, CH_3_CH_2_OH, Supelco, Sigma-Aldrich, Merck Inc., Philippines), nanoclay montmorillonite (Surface modified 35–45 wt.% dimethyl dialkyl (C14–C18) amine, Sigma-Aldrich, Merck Inc., Philippines), and distilled water (Nature Spring, Mandaue City, Philippines). The chemicals were used without further purification.

### 2.1. Acetylation Treatment

Every 1 g of ≤1 mm maguey cellulose (MC) was measured using the analytical balance and acetylated using the acetic anhydride reagent at 10 and 15 mL, under various stand times (16, 24, and 32 h). The acetylation solution was carried out in a 100 mL beaker at room temperature with 40 mL of glacial acetic acid. The acetic anhydride reagents are poured consecutively and mixed using a glass stirring rod, followed by the gradual addition of 6 drops of the sulfuric acid ($H_{2}{SO}_{4}$) catalyst, and stirred using a glass stirring rod. MC was transferred carefully into a 250 mL volumetric flask using a tweezer, and the acetylation solution from the beaker was carefully poured into the flask. The flask was sealed further using a paper masking tape to prevent the pungent smell from leaking. The samples were then left under various stand times at 16, 24, and 32 h. After achieving the various stand times, the acetylated samples were washed using distilled water and filtered using a cheesecloth. The washing and filtration process was repeated until the filtrate reached pH 7. The samples were then oven-dried at 80 °C for 3 h. The final products, maguey cellulose acetate (MCA), were then stored and sealed into a Ziploc bag for further use, investigation, and analysis. From the obtained maguey cellulose acetate, the MCA yield ($Y_{MCA}$) was calculated using Equation (1) as follows:

(1) $$\%\;Cellulose\;Acetate\;Yield\;(Y_{MCA})\;=\;\frac{m_{MCA}}{m_{MC}}\times 100$$

where $Y_{MCA}$ is the maguey cellulose acetate yield, $m_{MCA}$ is the mass of maguey cellulose acetate (g), and $m_{MC}$ is the mass of MC (g).

### 2.2. Bioplastic Fabrication

**Figure 1:**
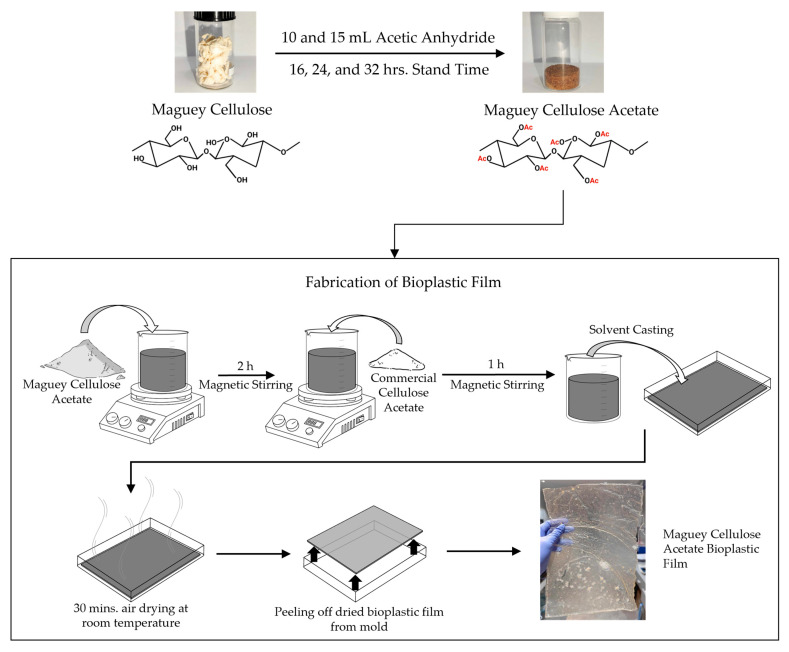
Schematic diagram of maguey cellulose acetate bioplastic film fabrication through the solvent casting method.

The bioplastic fabrication using maguey cellulose is depicted in Figure 1 below. A bioplastic solution was prepared using 6% (w/v) of MCA in acetone. A total of 6 g of MCA was added to a 250 mL beaker containing 100 mL of acetone (3:50 g/mL ratio) [3,35]. At room temperature, the MCA bioplastic solution was stirred using a magnetic stirrer at 300 rpm for 3 h or until completely dissolved. During the stirring process of the solution, the beaker’s mouth was sealed with aluminum and taped at the sides to prevent rapid evaporation of the solvent. The MCA bioplastic solution was then solvent casted into an A4-sized glass mold. The MCA bioplastic solution was air-dried at room temperature for 30 min or until the bioplastic film completely lifted itself from the glass mold. Once dried, the bioplastic solution was coagulated using pure ethanol for 30 min [35,36]. After drying the bioplastic, it was stored in a clean plastic envelope to avoid sudden shrinking. Additionally, various concentrations of commercial cellulose acetate (CCA) at 0, 15, 25, 35, and 50% (w/w) were added into the 6% (w/v) MCA bioplastic solution that was studied.

### 2.3. Incorporation of Nanoclay into Maguey Cellulose Acetate Bioplastic

Nanoclay at 0.5, 1, and 3% (w/w) was prepared and mixed into the 15% (w/w) CCA-MCA bioplastic solution. MCA was measured using the analytical balance and then poured into a beaker containing 100 mL of acetone solvent. MCA was stirred using the magnetic stirrer at 300 rpm for two (2) h. While the solution was being stirred, 15% (w/w) CCA was added and continuously stirred for an additional 1 h. Afterwards, nanoclay montmorillonite at 0.5, 1, and 3% (w/w) was measured using the analytical balance and slowly added into the 15% (w/w) CCA-MCA bioplastic solution, creating an NC-CCA-MCA bioplastic solution. NC-CCA-MCA was then magnetically stirred for 30 min, ultrasonicated for 30 min, and magnetically stirred again for 30 min to prevent sample settling.

### 2.4. Characterization

#### 2.4.1. Fourier-Transform Infrared Spectroscopy (FTIR) of Maguey Cellulose Acetate

FTIR analysis for the maguey cellulose acetate samples was performed using the IRAffinnity-1s, SHIMADZU, Kyoto, Japan, through zinc selenide attenuated total reflectance (ZnSe, ATR, SHIMADZU, Kyoto, Japan) in the range of 4200 to 1200 ${cm}^{-1}$. The samples were ground into a fine powder using an electric powder grinder before the analysis.

#### 2.4.2. Morphological Analysis via Scanning Electron Microscopy (SEM)

SEM analysis for the maguey fibers, maguey cellulose, maguey cellulose acetate, MCA bioplastic films, and nano-incorporated MCA bioplastic films was analyzed using the JEOL JCM 7000, JEOL Ltd., Tokyo, Japan at 10.0 kV. The samples were coated with a gold coat using the DII-29030SCTR Smart Coater, JEOL Ltd., Tokyo, Japan, for approximately 1 to 2 min.

#### 2.4.3. Tensile Strength of MCA Bioplastic Films via the Universal Testing Machine (UTM)

A total of 0.5, 1, and 3% NC to 15% (w/w) CCA-MCA bioplastic films were measured for thickness using a digital caliper. The tensile strength of NC-MCA-CCA bioplastic films was analyzed using the HC-301 Single Column Electric Digital Pull Force Testing (Laryee Technology Co., Ltd., Beijing, China), in reference to the ASTM standard method D882 [37]. The originally A4-sized films were cut into 100 mm by 25 mm uniform strips. The gauge length measured 50 mm, and the grip measurement was 25 mm on both the left and right sides. Triplicate samples were measured.

#### 2.4.4. Water Vapor Transmission Rate (WVTR) of MCA Bioplastic Films

The water vapor transmission rate (WVTR) of the film was measured using the desiccant method, following the ASTM E96/E96M standard [38]. Petri dishes containing anhydrous CaCl_2_ were sealed with the film samples and placed under controlled laboratory conditions maintained at 65 ± 5% relative humidity and 25.6 ± 0.5 °C. The Petri dishes were weighed at one-hour intervals, recording a total of nine measurements. The obtained weights were plotted against time, and the slope of the resulting line—calculated using the least squares regression method—represented the average rate of weight change (*G*/*t*). The water vapor transmission rate (WVTR) of the film samples was then calculated using Equation (2) as follows:

(2) $$WVTR=(\frac{G}{t})(\frac{1}{A})$$

where *A* denotes the test area. Triplicate samples were measured.

#### 2.4.5. Water Contact Angle Analysis (WCA) via an Optical Tensiometer

WCA analysis for the maguey cellulose acetate was analyzed using the Attension ThetaFlex Contact Angle Meter (Biolin Scientific AB, Gothenburg, Sweden), with water as the dispensing solvent. The MCA powdered samples were placed flat in the center of the microscopic slides. Triplicate samples were measured.

## 3. Results and Discussion

### 3.1. Synthesis of Maguey Cellulose Acetate by Acetylation

#### 3.1.1. Cellulose Acetate Yield and Degree of Substitution

The 10 mL and 15 mL acetic anhydride concentrations were under 16, 24, and 32 h. MCA presented the highest yield of 81.34% after acetylation treatment at 10 mL acetic anhydride under a 32 h acetylation stand time. Additionally, all MCA samples showed more than 70% yield, as shown in Table 1.

The final maguey cellulose acetate (MCA) was a brownish powder. Acetylation is typically governed by reagent concentration, stand time, and temperature [39]. Here, acetylation is conducted at room temperature for better and practical control, focusing on the effects of concentration and stand time. A key finding was that a longer acetylation stand time yielded a higher acetate percentage. Specifically, the MCA treated with 10 mL acetic anhydride for 32 h showed the highest yield at 81.34%, followed by the 15 mL treatment for 32 h at 76.86%. This aligns with the literature, suggesting that increased acetylation time elevates the maximum acetyl percentage, leading to a higher yield [40,41]. Both acetic anhydride concentration and stand time influence acetylation; however, simultaneously using higher concentrations and longer stand times may risk the disintegration of the cellulose acetate material. Notably, the MCA produced from locally sourced maguey cellulose demonstrated a competitive yield compared to that reported for other bio-raw materials, like rice husks, palm trees, rice straw, and recycled straw, which range from 66% to 94% [42,43,44,45,46,47].

The calculated degree of substitution (DS) based on the FTIR, for CCA and MCA, is 2.55 and 1.90, respectively. The blend used in this study represents a strategic combination of two distinct polymers to achieve balanced packaging properties. The CCA (DS 2.55), which sits at the upper end of the diacetate range, provides the blend with greater intrinsic hydrophobicity and higher mechanical strength compared to the lower DS MCA. Conversely, the MCA (DS 1.9) provides a source of cost-effective, renewable material and contributes to the bioplastic’s biodegradability due to its higher number of residual hydroxyl groups, which also slightly increases the polarity of the blend. By combining the two at a 15% CCA/MCA ratio, the researchers were able to fabricate bioplastic films that leverage the superior barrier and strength-imparting qualities of the higher DS CCA while maximizing the utilization of the newly synthesized, sustainable MCA.

#### 3.1.2. Fourier-Transform Infrared (FTIR) Spectroscopy Analysis of Maguey Cellulose Acetate and Commercial Cellulose Acetate

**Figure 2:**
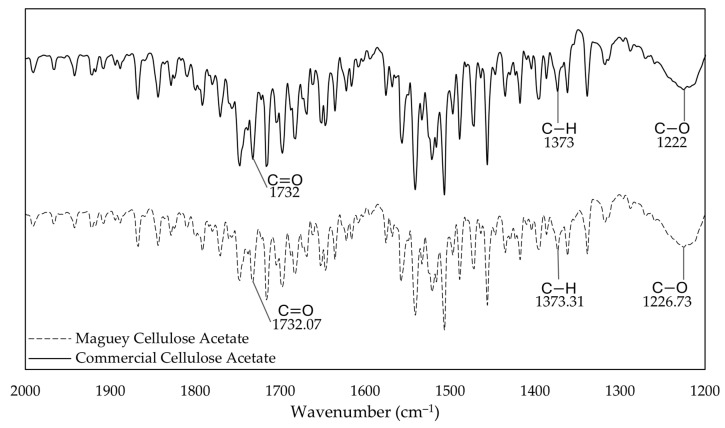
Fourier-transform infrared spectroscopy (FTIR) spectrum of maguey cellulose acetate and commercial cellulose acetate. (Data were generated using Microsoft Excel).

The results of the FTIR analysis show significant peaks of the infrared transmittance spectrum of maguey cellulose acetate and commercial cellulose acetate, as presented in Figure 2. The figure shows functional acetyl groups commonly navigated within the range of 2000 to 1200 ${cm}^{-1}$ that were found to be present in both MCA and CCA.

The maguey cellulose acetate showed peaks at 1732.07 ${cm}^{-1}$, indicating the presence of the C=O ester functional group and confirming the chemical conversion of the cellulose acetate ester, where 1373.31 ${cm}^{-1}$ represents C–H bending and 1226.73 ${cm}^{-1}$ signifies the C–O stretch commonly found within acetyl functional groups. CCA was used as a baseline for FTIR analysis of MCA, which confirmed the resemblance of important functional peaks, where the C=O peak is present at 1732 ${cm}^{-1}$, the C–H bend is at 1373 ${cm}^{-1}$, and the C–O stretch was aligned at 1222 ${cm}^{-1}$. Almost similar value ranges were found in a study on the acetylation of the Date Palm Trunk, where the C=O stretching of the acetyl group was located at ≈1740–1732 ${cm}^{-1}$, the C–H bending vibrations were at ≈1369–1365 ${cm}^{-1}$, and C=O stretching was found at ≈1222–1215 ${cm}^{-1}$ [44]. A quantitative study on cotton linters cellulose acetate also gave a significantly similar result, finding C=O acetyl stretching at 1750 ${cm}^{-1}$, followed by C–H vibrations at 1370 ${cm}^{-1}$ and lastly, C–O stretching at 1240 ${cm}^{-1}$ [48].

#### 3.1.3. Surface Morphology of Maguey Fibers, Maguey Cellulose, and Maguey Cellulose Acetate

**Figure 3:**
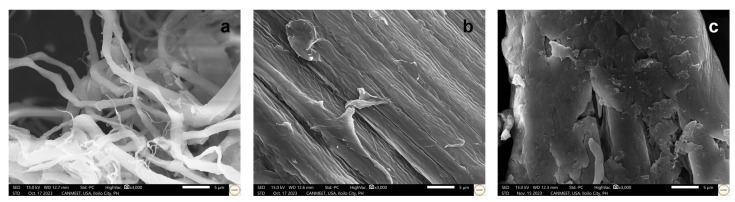
Morphological structure of (**a**) maguey fibers, (**b**) maguey cellulose, and (**c**) maguey cellulose acetate. Scale bars are all in 5 microns.

The SEM micrographs of the maguey fibers, maguey cellulose, and maguey cellulose acetate under 3000× magnification are presented in Figure 3. The following image analyses show the morphological change in structure from the fibers to the acetate form. The various treatments involved were alkali and bleaching treatment [9] and acetylation treatment.

The initial maguey fibers (as shown in Figure 3a) exhibited a tangled, strand-like structure with irregular sizes, a result of the heavy presence of impurities, lignin, and other extractives within the outer cellulose wall. A significant morphological difference is evident in Figure 3b, which displays the maguey cellulose (MC) as having a clear cellulose wall and a parallel structure, indicating the successful removal of impurities. The morphology of the resulting maguey cellulose acetate (MCA), presented in Figure 3c, closely resembles MC but appears more porous and granulated with a slightly exposed inner layer. Unlike a similar study on chestnut starch acetylation, where the original material was smooth, our maguey cellulose initially appeared smooth without cracks, but acetylation resulted in noticeable concave pits or perforations [49]. The perforated areas observed in the MCA morphology expose portions of the inner cylindrical fibers, which could be attributed to the swelling of cellulose hydroxyl groups that facilitates the conversion to acetyl groups [50,51]. This structure, featuring a surface with cylindrical fibers and perforations, is similar to the morphology described in the acetylation of Oil Palm Empty Fruit Bunches Cellulose [52].

#### 3.1.4. Surface Morphology of Maguey Cellulose Acetate and Commercial Cellulose Acetate

**Figure 4:**
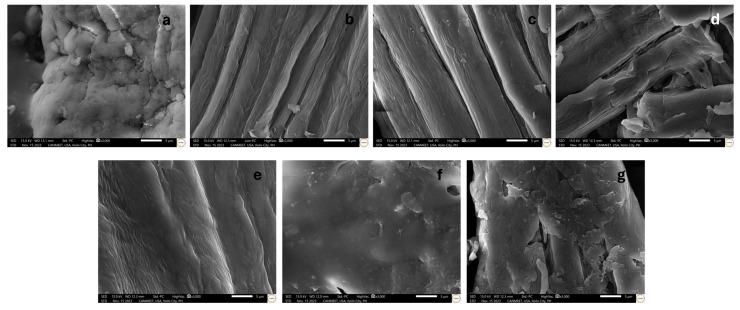
Morphological structure of (**a**) commercial cellulose acetate, (**b**) 10 mL acetic anhydride concentration under 16 h acetylation stand time, (**c**) 10 mL acetic anhydride concentration under 24 h acetylation stand time, (**d**) 10 mL acetic anhydride concentration under 32 h acetylation stand time, (**e**) 15 mL acetic anhydride concentration under 16 h acetylation stand time, (**f**) 15 mL acetic anhydride concentration under 24 h acetylation stand time, and (**g**) 15 mL acetic anhydride concentration under 32 h acetylation stand time. Scale bars are all in 5 microns.

Figure 4 presents the morphological structure of maguey cellulose acetate (MCA) produced using 10 mL and 15 mL of acetic anhydride at 16, 24, and 32 h of acetylation stand time, with commercial cellulose acetate (CCA) (Figure 4a) serving as the morphological baseline. Treatment with 10 mL of acetic anhydride for 16 h (Figure 4b) resulted in MCA with little to no structural change compared to raw maguey cellulose (MC) (Figure 3c), exhibiting a smooth surface that lacked the porosity, indentations, and granular appearance of the CCA baseline. Increasing the stand time to 24 h (Figure 4c) showed a notable difference, with porous areas and indentations beginning to form. At 32 h (Figure 4d), the MCA morphology closely resembled the baseline CCA, distinctly exhibiting porous and granulated areas, which morphologically implicates the successful permeation of the acetylation treatment. In contrast, the 15 mL treatment for 16 h (Figure 4e) produced a parallel and uniform structure, although not all parallel strands had a uniform diameter. Extending the 15 mL treatment to 24 h (Figure 4f) maintained a similar uniform-diameter and parallel strand morphology but featured a textured and fibrous surface, rather than the porous, granulated structure of the CCA baseline. Finally, the 15 mL treatment at 32 h (Figure 4g) resulted in a fragmented and disoriented morphological structure, suggesting that the strong penetration achieved by the high concentration and long duration likely caused disintegration.

#### 3.1.5. Water Contact Angle of Commercial Cellulose Acetate and Maguey Cellulose Acetate

**Figure 5:**
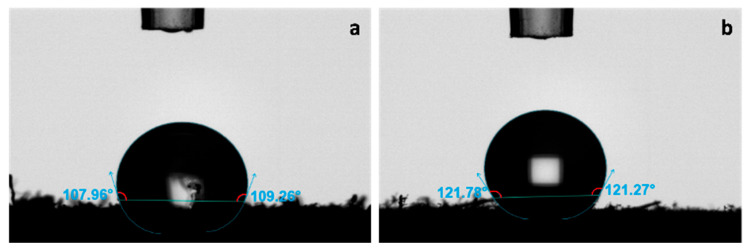
Water contact angle wettability analysis of (**a**) commercial cellulose acetate and (**b**) maguey cellulose acetate.

The water contact angle (WCA) analysis was conducted on both commercial cellulose acetate (CCA) and maguey cellulose acetate (MCA), as presented in Figure 5. The MCA treated with 10 mL of acetic anhydride for 32 h was selected for this investigation because it showed significant acetyl functional groups via infrared spectroscopy and exhibited the closest morphological resemblance to the baseline CCA. The baseline CCA (Figure 5a) recorded a WCA of 108.671°, confirming the material’s hydrophobicity. The MCA (Figure 5b) showed an even greater hydrophobic value of 121.897°. While neat cellulose is inherently hydrophilic due to its hydroxyl groups [53], the acetylation process successfully converts these hydrophilic groups into hydrophobic acetyl groups in both the CCA and MCA. Thus, both powdered acetates exhibited hydrophobic wettability, with contact angles significantly greater than 90°.

### 3.2. Fabricated Bioplastic from Maguey Cellulose Acetate

#### 3.2.1. Characterization of MCA Bioplastic Film

**Figure 6:**
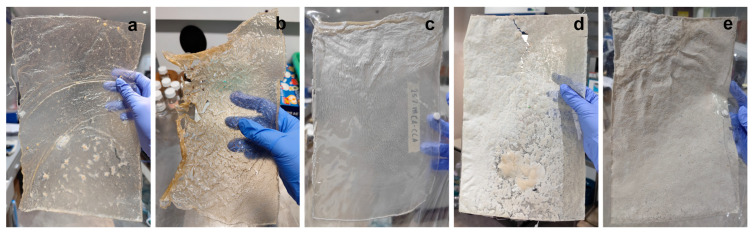
Physical appearance of bioplastic film having (**a**) 0% CCA, (**b**) 15% CCA, (**c**) 25% CCA, (**d**) 35% CCA, and (**e**) 50% CCA (w/w) concentrations at a 6% (w/v) ratio of the MCA–acetone bioplastic solution.

Pure MCA bioplastic film, by itself, is not the most suitable for nano-incorporation because of its low mechanical properties. Within this study, the bioplastic solution utilizes a 6% (*w*/*v*) MCA–acetone solution. The degree of substitution of acetone-soluble CCA is stable at the diacetate level. CCA was added to further solidify the structure of the MCA bioplastic film. This section investigated the most suitable CCA concentration (15, 25, 35, and 50% (*w*/*w*)) for bioplastic fabrication in terms of physical attributes, morphology, and wettability. Characterization was performed on A4-sized MCA bioplastic film having CCA concentrations at 0, 15, 25, 35, and 50% (w/w) into a 6% (w/v) ratio of the MCA–acetone bioplastic solution. The physical appearance of MCA and CCA-MCA bioplastics films can be found in Figure 6.

A key advantage of cellulose acetate (CA) is its inherent transparency during fabrication [54]. The physical appearance of the resulting films, shown in Figure 6, clearly changes with increasing commercial cellulose acetate (CCA) concentration. The 0% CCA film (Figure 6a) is transparent, though it exhibits minor agglomeration due to incomplete phase transition of the maguey cellulose acetate (MCA) into the solution. This could be remedied with longer magnetic stirring. The 15% CCA film (Figure 6b) retains similar transparency to the 0% film, with its subtle brown color attributed to the MCA powder. However, wrinkling suggests a structural flaw, possibly caused by over-drying or improper storage. The 25% CCA film (Figure 6c) shows decent fabrication but is notably opaque rather than transparent. Figure 6d presents severe issues, with obvious surface agglomeration leading to cracks and fissures, indicating that the components did not blend well. Given the susceptibility of CCA and MCA, which have varying sizes and micro-structures, to agglomerate within the polymer matrix [55], the ratio and concentration are critical fabrication factors. Finally, the 50% CCA film (Figure 6e) is evidently opaque and brittle, making it unsuitable for bioplastic fabrication. Despite lacking a major agglomeration, dispersed tiny clumps are physically present, highlighting the poor outcome of a large CCA-MCA ratio.

#### 3.2.2. Surface Morphology of CCA-MCA Bioplastic Films

**Figure 7:**
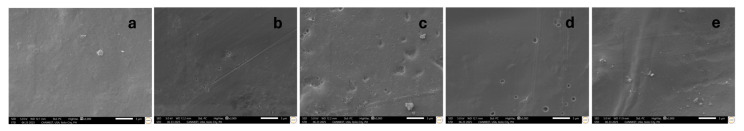
Surface morphology of bioplastic films having (**a**) 0% CCA, (**b**) 15% CCA, (**c**) 25% CCA, (**d**) 35% CCA, and (**e**) 50% CCA (w/w) concentrations at a 6% (w/v) ratio of the MCA–acetone bioplastic solution. Scale bars are all in 5 microns.

The surface morphology of the CCA-MCA films was studied to observe the surface characteristics of the films and the interaction and dispersion between CCA and MCA. The SEM micrographs of the CCA-MCA fabricated bioplastic films are presented in Figure 7 using 3000× magnification.

The surface morphologies of the commercial cellulose acetate–maguey cellulose acetate (CCA-MCA) films, viewed at 3000× magnification, revealed key structural differences. Figure 7a, representing the 0% CCA film, shows a subtly textured surface with minor indentations, likely due to the inherent brittleness of micro-sized cellulose in the bioplastic. Figure 7b exhibits a generally smooth morphology with mild lines, which may be due to mechanical stress from gradual shrinking during ambient temperature drying, as no human intervention occurred. Slight porous pits were also observed, which can contribute to low mechanical strength and brittleness. Figure 7c is relatively similar to Figure 7b but contains large indentations and scattered small pits, suggesting structural flaws that compromise mechanical properties. Figure 7d presents a clear and compact surface, though the presence of deep holes could compromise the film’s quality and integrity. Figure 7e also exhibits a smooth and compact surface, but irregular indentures and wrinkling are visible, possibly caused by uneven dispersion of CCA and MCA in the solution or premature drying of the exposed top layer. Based on these observations, the 15% CCA-MCA bioplastic films were deemed the most suitable candidates for nanoclay incorporation due to their smooth surface morphology and minimal flaws.

#### 3.2.3. Water Contact Angle of CCA-MCA Bioplastic Films

**Figure 8:**
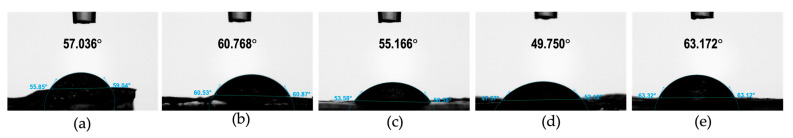
Water contact angle of (**a**) 0% CCA, (**b**) 15% CCA, (**c**) 25% CCA, (**d**) 35% CCA, and (**e**) 50% CCA (w/w) concentrations at a 6% (w/v) ratio of the MCA–acetone bioplastic solution.

The wettability of the CCA-MCA films was studied to observe the interaction of water with the bioplastic film surface and to identify whether the material is hydrophobic or hydrophilic. The water contact angles of the CCA-MCA fabricated bioplastic films are presented in Figure 8.

The wettability of the cellulose acetate–maguey cellulose acetate (CCA-MCA) films is a key factor in determining their suitability for packaging applications [56]. All MCA bioplastic films, across 0%, 15%, 25%, 35%, and 50% CCA concentrations, exhibited hydrophilic wettability, with water contact angle (WCA) values of less than 90°, consisting of 57.036°, 60.768°, 55.166°, 49.750°, and 63.172°, respectively. Although the 50% (*w*/*w*) CCA-MCA film (Figure 8e) had the highest WCA of 63.172°, its opaque physical appearance and wrinkled surface morphology rendered it unsuitable. Therefore, the second-highest value, 60.768°, found in the 15% (*w*/*w*) CCA-MCA bioplastic film (Figure 8b), was deemed the most appropriate for subsequent nanoclay (NC) incorporation. These results are comparable to the WCA values of 60° to 70° of bioplastics derived from *Prosopis juliflora* cellulose, though they are higher than the relatively low hydrophilic contact angle of 47.25° observed in corn starch bioplastic films [57,58].

### 3.3. Fabricated Bioplastic from Nano-Incorporated Maguey Cellulose Acetate

#### 3.3.1. Characterization of Nano-Incorporated MCA Bioplastic Film

Characterization was performed for the MCA bioplastic film with a 15% (*w*/*w*) CCA constant and NC concentrations, as shown in Table 2.

#### 3.3.2. Mechanical Properties of the Nano-Incorporated MCA Bioplastic Films

**Figure 9:**
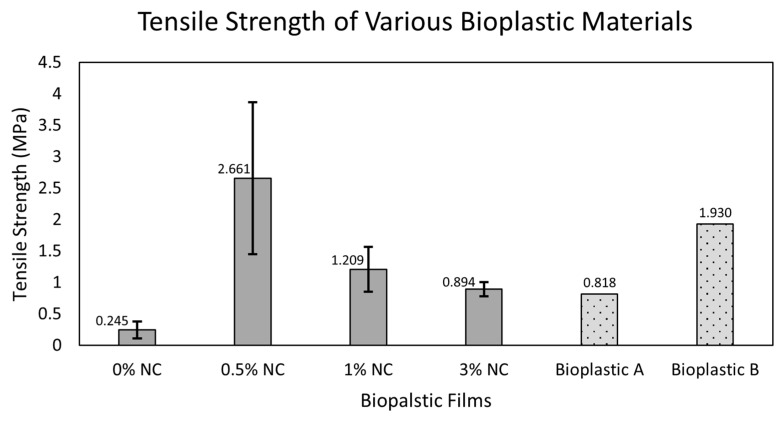
Tensile strength of 0% NC at 15% (*w*/*w*) CCA-MCA bioplastic, 0.5% NC at 15% (*w*/*w*) CCA-MCA, 1% NC at 15% (*w*/*w*) CCA-MCA, 3% NC at 15% (*w*/*w*) CCA-MCA, and the best tensile results from related research journals of bioplastic film fabrications. Kapok CA with glycerol plasticizer; CNC bioplastic from mangosteen peel with glycerol.

Determining the mechanical property is an important factor in revealing the polymer material’s mechanical behavior and determining whether the material is suitable for its intended purpose [59]. The tensile strength results of 0.5, 1, and 3% NC-incorporated bioplastic films and the baseline, 15% (*w*/*w*) CCA-MCA bioplastic, are presented in Figure 9. Additionally, the highest tensile strength from related studies, such as the cellulose acetate synthesized from Kapok [60] bioplastic films and cellulose nanocrystal (CNC)-reinforced bioplastic from mangosteen, was identified for reference [23]. The baseline bioplastic film showed a low tensile strength of 0.245 MPa; this is possibly because the baseline bioplastic film has no addition of any form of plasticizer or filler, further solidifying that cellulose-based bioplastic by itself has low mechanical strength. The highest tensile strength was found from the 0.5% NC at 15% (*w*/*w*) CCA-MCA bioplastic with 2.0112 MPa, followed by 1% NC at 15% (*w*/*w*) CCA-MCA bioplastic with a value of 0.8862 MPa. Lastly, 3% NC at 15% (*w*/*w*) CCA-MCA bioplastic presented a value of 0.777 MPa.

##### Welch’s *t*-Test of Mechanical Properties of the Nano-Incorporated MCA Bioplastic Films

Three independent Welch’s *t*-tests were performed to compare the tensile strength of the baseline film (0% NC) against the three nanoclay concentrations. Bonferroni correction was applied to decrease the chance of a false positive (Type 1) error. With three tests (m = 3), the Bonferroni corrected significance threshold (*a_new_*) is (0.053) = 0.01667. The *p* values of 0.5% NC, 1% NC, and 3% NC are as follows: 0.0727, 0.0314, and 0.0032.

There is a significant improvement in tensile strength when compared to the baseline 15% (*w*/*w*) CCA-MCA bioplastic film (0.245 MPa) and the 0.5% NC film (2.661 MPa), respectively. However, the statistical analysis indicates that only the decrease in tensile strength observed at the 3% NC concentration (0.894 MPa) is statistically significant compared to the 0% NC control (0.245 MPa). This significant change confirms a real effect of the nanoclay addition. It is also observed that from 0.5% NC incorporation up to 3% incorporation, there is a declining trend for the tensile strength (2.661 MPa, 1.209 MPa, 894 MPa). Thus, while nanoclay assists in enhancing mechanical properties, large amounts of nanoclay addition cause a compromise to the material and create a brittle mechanical characteristic. The 0.5%, 1%, and 3% (*w*/*w*) nanoclay concentrations at 15% CCA-MCA bioplastic films showed comparable results to related studies. The Kapok CA bioplastic with glycerol plasticizer resulted in a 0.818 MPa tensile strength, which is competitively similar to the 1% NC-incorporated bioplastic film (1.209 MPa). The tensile strength range of 0.894 MPa to 2.661 MPa from 3% and 0.5% NC, respectively, shows a competitive range of tensile strength compared to the 0.818 MPa tensile strength from the Kapok CA bioplastic with glycerol plasticizer. While the 2.661 MPa tensile strength from 0.5% NC at 15% (*w*/*w*) CCA-MCA bioplastic is comparably competitive to the 1.93 MPa tensile strength of mangosteen-synthesized CNC-reinforced bioplastic.

#### 3.3.3. Water Vapor Transmittance Rate of the Nano-Incorporated MCA Bioplastic

**Figure 10:**
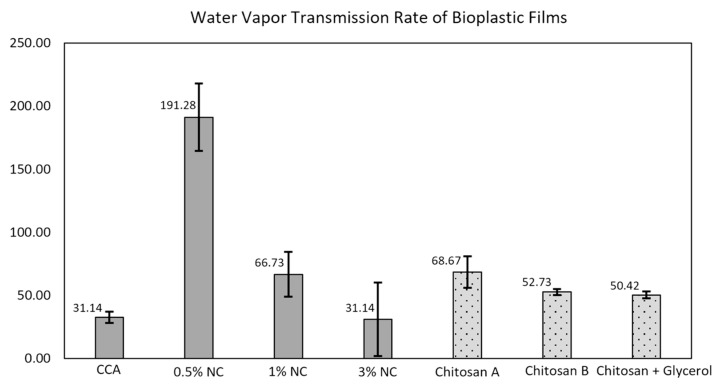
Water vapor transmission rate of nano-incorporated 15% (*w*/*w*) CCA-MCA bioplastic films and biomass-based bioplastic films from related studies. Chitosan A is based on Zhong et al.’s study [61], Chitosan B is from Xu et al.’s study [62], and chitosan + glycerol is from Amaba et al.’s study [63].

The water vapor transmittance rate was investigated to study the barrier quality of the 0.5, 1, and 3% NC-incorporated bioplastic films, as well as the baseline, 15% (*w*/*w*) CCA-MCA bioplastic, which is presented in Figure 10. Conversely, the vapor transmittance rate of bioplastics from the studies of Zhong et al. [61], Xu et al. [62], and Amaba et al. [63] was identified for reference. We identified the quality barrier properties that serve in the prevention of packaging deterioration and that are commonly caused by environmental factors such as moisture, vapor, and heat [63,64]. Among all samples, the CCA and 3% NC films exhibited the lowest WVTR values (31.14 g/m^2^·h), indicating superior resistance to water vapor transmission. Conversely, the 0.5% NC film displayed a markedly higher WVTR (191.28 g/m^2^·h), suggesting that excessive or uneven dispersion of nanocellulose can increase the film’s permeability. In comparison with the literature values for pure chitosan and bioplastic films, the CCA and 3% NC formulations demonstrated enhanced barrier performance, making them promising candidates for moisture-sensitive packaging or coating applications. The relationship between a bioplastic’s Water Vapor Transmission Rate (WVTR) and the composition of the nanomaterial used is generally an inverse relationship, where the correct incorporation of nanomaterials significantly reduces the WVTR by creating a tortuous path for water molecules to diffuse. Nanomaterials with a high aspect ratio (e.g., layered nanoclays or needle-shaped cellulose nanocrystals) are impermeable to water molecules. When they are uniformly dispersed and aligned perpendicular to the film surface, they force water vapor molecules to travel a much longer, zigzag path through the polymer matrix to pass from one side of the film to the other [65]. A study on the mechanical properties of nanoclay-incorporated bio-based packaging material described that with 10 wt.% montmorillonite nanoclay, the tensile strength increased, while its water barrier film properties decreased [66].

##### Welch’s *t*-Test of Water Vapor Transmittance Rate of the Nano-Incorporated MCA Bioplastic Films

Three independent Welch’s *t*-tests were performed to compare the WVTR of the baseline film (0% NC) against the three nanoclay concentrations (0.5% NC, 1% NC, and 3% NC). Bonferroni correction was applied to decrease the chance of a false positive (Type 1) error. With three tests (m = 3), the Bonferroni corrected significance threshold (*a_new_*) is (0.053) = 0.01667. The *p* values of 0.5% NC, 1% NC, and 3% NC are as follows: 0.00779, 0.06647, and 1.000.

The analysis revealed that the increase in WVTR at the 0.5% NC concentration was statistically significant (*p* = 0.00779). This finding supports the hypothesis that the nanoclay at this low ratio is likely unevenly dispersed or aggregated, leading to defects in the film’s morphology that severely compromise its barrier performance, resulting in a reliably higher WVTR (191.28 g/m^2^·h). Conversely, the differences observed for the 1% NC (*p* = 0.06647) and 3%NC (*p* = 1.0000) concentrations were not statistically significant against the CCA baseline (31.14 g/m^2^·h). The non-significance of the 1% NC group is likely due to high data variability (STDEV of 17.79), while the 3% NC group’s non-significance confirms that its mean WVTR is virtually identical to the control, demonstrating that the higher nanoclay concentration successfully returned the barrier properties to the baseline level, though it did not achieve a statistically significant improvement.

#### 3.3.4. Surface Morphology of the Nano-Incorporated MCA Bioplastic Films

**Figure 11:**
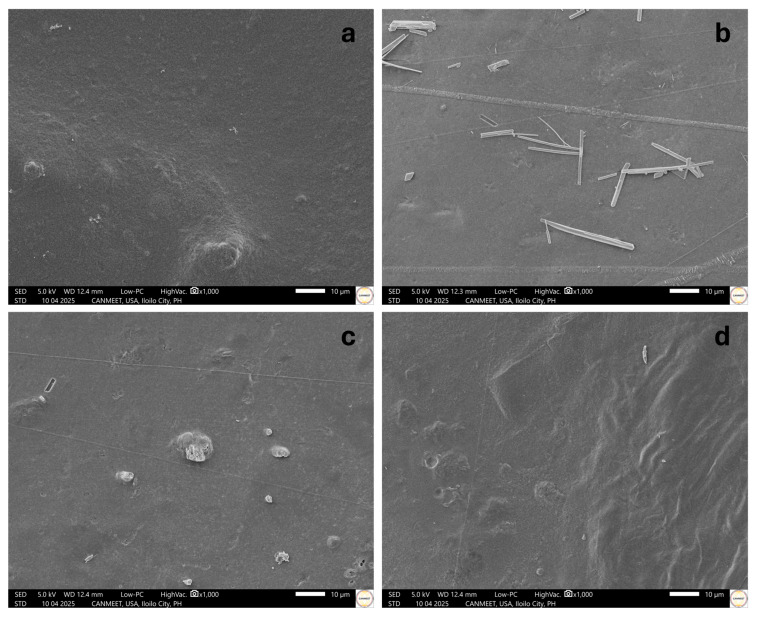
Surface morphology of (**a**) baseline 15% (*w*/*w*) CCA-MCA bioplastic film, (**b**) 0.5% NC at 15% (*w*/*w*) CCA-MCA bioplastic film, (**c**) 1% NC at 15% (*w*/*w*) CCA-MCA bioplastic film, and (**d**) 3% NC at 15% (*w*/*w*) CCA-MCA bioplastic film. Scale bars are all in 10 microns.

The surface morphology of the films was studied to observe the characteristics of the films and the dispersion of nanoclay (NC) within the commercial cellulose Acetate–maguey cellulose acetate (CCA-MCA) matrix. The Scanning Electron Microscopy (SEM) micrographs of the nano-incorporated bioplastic films at 1000× magnification are presented in Figure 11. In Figure 11a, the baseline 15% (*w*/*w*) CCA-MCA film shows a rough and bumpy texture with scattered tiny aggregates and micro hill-like structures, suggesting a partially dissolved bioplastic solution. This rough, bumpy surface morphology, without nanomaterial incorporation, is indicative of brittleness and poor tensile strength [58]. Upon adding NC, the morphology changed. Figure 11b (0.5% NC) exhibits a smooth and homogeneous surface overall, though several fiber-like structures were present, possibly from incomplete dispersion of MCA in the NC-CCA-MCA solution. The observed micro-fissure ridge may be caused by an interfacial boundary formed between the swollen MCA and the matrix [59]. Figure 11c (1% NC) shows a flawed yet compact surface, with small holes, dents, and protrusions likely resulting from incomplete dispersion of NC-CCA-MCA within the matrix. Finally, Figure 11d (3% NC) displays an unsmooth, wrinkly, and textured surface. This wrinkling characteristic can be attributed to the slow drying of the high-concentration 3% NC bioplastic solution; as the exterior layer shrinks while the interior is still drying, the surface morphology becomes deformed.

**Figure 12:**
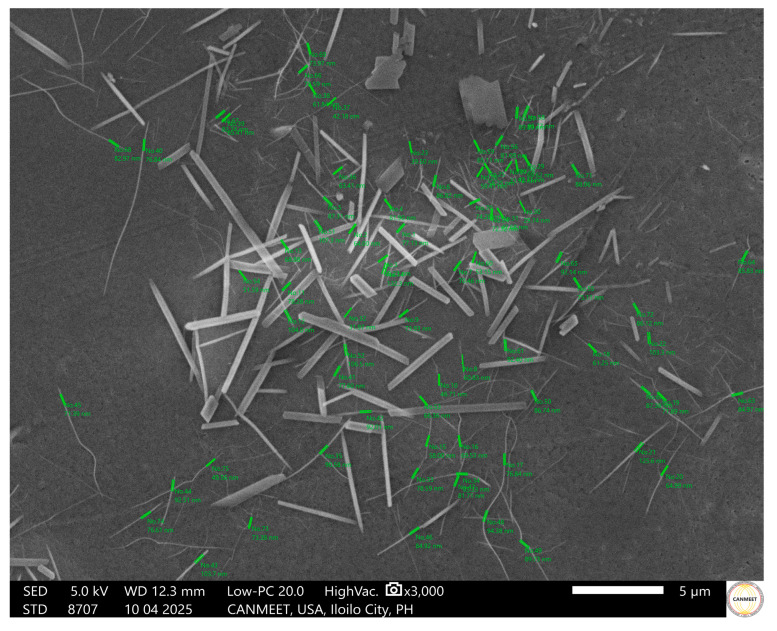
Surface morphology of 0.5% NC at 15% (*w*/*w*) CCA-MCA bioplastic film. Scale bar is in 5 microns.

The surface morphology of the 0.5% NC at 15% (*w*/*w*) CCA-MCA bioplastic film was observed at 3000× magnification in Figure 12. Observing the morphological structure of the NC-CCA-MCA bioplastic film is significant for understanding how the incorporated nanoclay materials behave as part of the film. This investigation at high magnification also reveals the effectiveness of nanoclay dispersion during the fabrication process and the size of the nanomaterial present.

The simple mixing via magnetic stirring does not guarantee an appropriate dispersion of the nanoclay to its nano-size. Interventions such as ultrasonication and high-pressure homogenization are necessary to ensure effective dispersion. Ultrasonication was utilized during the bioplastic solution preparation for 30 min, as discussed within the Materials and Methods, specifically under Section 2.3. Incorporation of Nanoclay into Maguey Cellulose Acetate Bioplastic. Nanocomposites, from nanoclay, were found at the 0.5% NC at 15% (*w*/*w*) CCA-MCA bioplastic film, signifying the effective distribution of nanoclay through ultrasonication. The presence of nanomaterial, as shown in Figure 12, indicates that nanoclay is integrated and filled into the bioplastic matrix, potentially improving the mechanical properties of the bioplastic film. The nanocomposites were identified with size ranges from 29.74 nm to 107.3 nm.

#### 3.3.5. Wettability of the Nano-Incorporated MCA Bioplastic Film

**Figure 13:**
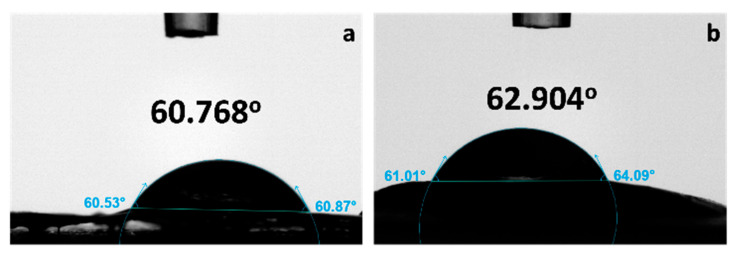
Water contact angle wettability of (**a**) 0% NC and (**b**) 0.5% NC at 15% (*w*/*w*) CCA-MCA bioplastic film.

The surface morphology of the baseline (0%) and 0.5% nano-incorporated bioplastic films at 15% (*w*/*w*) CCA-MCA bioplastic films was studied to compare and contrast the difference between cellulose acetate-based bioplastic film and nano-incorporated bioplastic film wettability. The water contact angles between 0% NC and 0.5% NC at 15% (*w*/*w*) CCA-MCA bioplastic films are presented in Figure 13.

The MCA bioplastic film with only 15% (*w*/*w*) CCA-MCA bioplastic film and no nanoclay incorporation has a wettability angle of 60.768° when compared with the 0.5% NC bioplastic film; for the 15% (*w*/*w*) CCA-MCA bioplastic film, there is a slight increase in the wettability, with an angle of 62.904°. The contact angle result of 0.5% nanoclay incorporation is not significantly higher than the baseline 15% (*w*/*w*) CCA-MCA bioplastic film. Despite NC montmorillonite being an effective composite in improving mechanical and barrier properties, this nanomaterial has disadvantages in terms of the dispersion method, as it requires modification [67]. Techniques and interventions, such as longer ultrasonication or high-pressure homogenization, are possible applications to improve nanomaterial dispersion for better water contact angle wettability and barrier properties. However, this very first attempt to successfully incorporate maguey fiber with a nanoclay additive in a CCA-MCA matrix. Given the novelty of this biocomposite system, our primary objective was to establish a clear proof of concept for the feasibility of creating a “nano-enabled bioplastic” using these specific components. Our chosen dispersion strategy was selected based on the established literature protocols for similar clay/polymer systems, allowing us to confidently achieve a baseline level of dispersion sufficient to observe and characterize the significant improvements in mechanical properties.

## 4. Conclusions

This study investigated the synthesis and characterization of maguey (*Agave cantala*) cellulose acetate (MCA) and the subsequent production of nano-incorporated bioplastic films. MCA was successfully synthesized, achieving an impressive yield of 81.34%, highlighting its potential for resource sustainability. Fourier-transform infrared spectroscopy (FT-IR) confirmed the key acetate functional groups in this sample: the C=O ester group at 1732.07 cm^−1^, the C-H bend at 1373.31 cm^−1^, and the C-O stretch at 1226.73 cm^−1^. The resulting MCA closely resembled commercial cellulose acetate (CCA) in morphology, appearing porous and granulated. Both the MCA and baseline CCA exhibited hydrophobic wettability, with water contact angles of 121.897° and 108.671°, respectively.

When fabricating bioplastic films, transparency shifted from transparent to opaque as the CCA concentration increased, ranging from the most transparent film (no CCA addition) to the fully opaque 50% CCA-MCA film. The 15% CCA-MCA film was selected for nano-incorporation due to its favorable properties: a smooth surface with minimal flaws (SEM analysis) and the second-highest water contact angle, 60.768°, among the bioplastic films. Nanoclay (NC) incorporation significantly enhanced the film’s mechanical properties, increasing the tensile strength from 0.245 MPa (baseline 15% CCA-MCA) to a competitive 2.0112 MPa at 0.5% NC concentration. However, a decreasing trend was observed, with higher NC concentrations leading to lower tensile strength. Conversely, the Water Vapor Transmission Rate (WVTR) showed a favorable effect with increased NC loading, dropping to a low of 31.14 g/m^2^-h at 3% NC compared to a higher value of 191.28 g/m^2^-h at 0.5% NC. Surface morphology at 1000× magnification showed that the 0.5% NC film had a smooth surface with minimal defects, indicating effective NC dispersion and filling; however, the presence of fiber-like structures prompted further analysis. At 3000× magnification, the 0.5% NC film confirmed the integration of nanocomposites, with nano-sized particles ranging from 29.74 nm to 107.30 nm. Finally, due to limited technical intervention in NC dispersion, the improvement in wettability between the 0% NC film (60.768°) and the 0.5% NC film (62.904°) was minimal.

While we acknowledge that the current material is not yet suitable for direct sachet replacement due to insufficient load-bearing capacity, the initial strength range (0.78–2.01 MPa) aligns well with applications that prioritize flexibility, low cost, and biodegradability over high mechanical strength. Potential immediate uses for materials in this early-stage range include (1) biodegradable agricultural films for low-strength films used for mulching, where high flexibility and environmental degradation are key requirements, and (2) compostable low-stress packaging, which includes very thin, single-use liners or wraps for non-critical, low-weight items (e.g., individual candies or extremely lightweight, non-load-bearing enclosures.) The immediate and critical next phase should involve comprehensive, multi-method validation of the degradation profile, such as soil burial or composting tests, to fully characterize the material’s environmental fate.

## Figures and Tables

**Table 1:** Yield of MCA after the acetylation method under 10 and 15 mL acetic anhydride concentrations at various acetylation stand times (16, 24, and 32 h).

Cellulose (g)	Acetic Anhydride (mL)	Acetylation Stand Time (h)	Weight After Drying (g)	Yield (%)
1.0136	10	16	0.7265	71.6725
1.0227	10	24	0.7373	72.0935
1.0183	10	32	0.8283	81.3415
1.0215	15	16	0.7338	71.8355
1.0128	15	24	0.7238	71.4652
1.0056	15	32	0.7729	76.8596

**Table 2:** Ratio of concentrations of nanoclay, commercial cellulose acetate, and maguey cellulose acetate for bioplastic film solutions.

Material	0.5% NC at 15% (*w*/*w*) CCA-MCA (g)	1% NC at 15% (*w*/*w*) CCA-MCA (g)	3% NC at 15% (*w*/*w*) CCA-MCA (g)
Nanoclay	0.0297	0.06	0.18
Commercial Cellulose Acetate	0.8995	0.891	0.873
Maguey Cellulose Acetate	5.0745	5.094	4.947

## Data Availability

The original contributions presented in this study are included in the article. Further inquiries can be directed to the corresponding author.
